# Complete genome determination and analysis of *Acholeplasma oculi* strain 19L, highlighting the loss of basic genetic features in the *Acholeplasmataceae*

**DOI:** 10.1186/1471-2164-15-931

**Published:** 2014-10-24

**Authors:** Christin Siewert, Wolfgang R Hess, Bojan Duduk, Bruno Huettel, Richard Reinhardt, Carmen Büttner, Michael Kube

**Affiliations:** Faculty of Life Science, Thaer-Institute, Division Phytomedicine, Humboldt-Universität zu Berlin, Lentzeallee 55/57, 14195 Berlin, Germany; Genetics & Experimental Bioinformatics, Institute of Biology III, University of Freiburg, Schänzlestraße 1, 79104 Freiburg, Germany; Institute of Pesticides and Environmental Protection, Banatska 31b, P.O. Box 163, 11080 Belgrade, Serbia; Max Planck Genome Centre Cologne, Carl-von-Linné-Weg 10, 50829 Cologne, Germany

## Abstract

**Background:**

*Acholeplasma oculi* belongs to the *Acholeplasmataceae* family, comprising the genera *Acholeplasma* and ‘*Candidatus* Phytoplasma’. Acholeplasmas are ubiquitous saprophytic bacteria. Several isolates are derived from plants or animals, whereas phytoplasmas are characterised as intracellular parasitic pathogens of plant phloem and depend on insect vectors for their spread. The complete genome sequences for eight strains of this family have been resolved so far, all of which were determined depending on clone-based sequencing.

**Results:**

The *A. oculi* strain 19L chromosome was sequenced using two independent approaches. The first approach comprised sequencing by synthesis (Illumina) in combination with Sanger sequencing, while single molecule real time sequencing (PacBio) was used in the second. The genome was determined to be 1,587,120 bp in size. Sequencing by synthesis resulted in six large genome fragments, while the single molecule real time sequencing approach yielded one circular chromosome sequence. High-quality sequences were obtained by both strategies differing in six positions, which are interpreted as reliable variations present in the culture population. Our genome analysis revealed 1,471 protein-coding genes and highlighted the absence of the F_1_F_O_-type Na^+^ ATPase system and GroEL/ES chaperone. Comparison of the four available *Acholeplasma* sequences revealed a core-genome encoding 703 proteins and a pan-genome of 2,867 proteins.

**Conclusions:**

The application of two state-of-the-art sequencing technologies highlights the potential of single molecule real time sequencing for complete genome determination. Comparative genome analyses revealed that the process of losing particular basic genetic features during genome reduction occurs in both genera, as indicated for several phytoplasma strains and at least *A. oculi*. The loss of the F_1_F_O_-type Na^+^ ATPase system may separate *Acholeplasmataceae* from other *Mollicutes*, while the loss of those genes encoding the chaperone GroEL/ES is not a rare exception in this bacterial class.

**Electronic supplementary material:**

The online version of this article (doi:10.1186/1471-2164-15-931) contains supplementary material, which is available to authorized users.

## Background

*Acholeplasma* species comprise bacteria of the family *Acholeplasmataceae* in the class *Mollicutes*, characterised by the lack of sterol requirement for growth and thereby separated from *Mycoplasmataceae* and *Spiroplasmataceae* [1]. The majority of *Acholeplasma* spp. are described as saprophytes and commensals. An evident assignment as pathogens is hampered by the fact that several *Acholeplasma* spp. are distributed ubiquitously. Moreover, no primary pathogen is described for this genus. However, the isolation of strains from diseased animals, and classification as putative animal pathogens, applies to species such as *A. axanthum* and *A. oculi* [2, 3]. This assignment of the type strain *A. oculi* 19L (syn. *A. oculusi*) was the result of its isolation from goat eyes infected with keratoconjunctivitis and re-infection experiments [2]. However, the assignment of *A. oculi* to this disease is rare in contrast to several *Mycoplasma* spp. [4].

Besides *Acholeplasma*, the *Acholeplasmataceae* family also includes the provisory taxon ‘*Candidatus* Phytoplasma’. Phytoplasmas are associated with several hundred plant diseases – and thus significant economic losses [5]. After insect vector-mediated transmission, phytoplasmas colonise as intracellular obligate parasites the sieve cells of a plant, often resulting in abnormal growth and reduced vitality. No general evidence for pathogenesis by acholeplasmas in colonised insects and plants has been provided to date. However, a recently published study on the *A. laidlawii* strain PG-8 supports its phytopathogenicity, which can be increased after nanotransformation in ultramicroform cells and might be correlated to extracellular vesicle formation under experimental conditions [6]. Further studies are needed in this respect, but the results may indicate a mechanism shared by both genera. In contrast, experimentally proven effector proteins or membrane proteins involved in phytoplasma-host interaction were not identified in the acholeplasmas [7]. These genetic elements of phytoplasmas might have originated from horizontal gene transfers. Massive gene loss, in combination with duplication events and genome instability, separates the phytoplasmas from the acholeplasmas. The complete genome sequences of eight strains of this family have been published, comprising the acholeplasmas *A. laidlawii* strain PG-8A [8], *A. brassicae* strain O502 and *A. palmae* strain J233 [7] and the phytoplasmas ‘*Ca*. P. australiense’ strain rp-A [9] and NZSb11 [10], ‘*Ca*. P. asteris’ strain OY-M [11] and AY-WB [12] and ‘*Ca*. P. mali’ strain AT [13]. In the past, all five phytoplasma strains and *A. laidlawii* were sequenced by applying the whole genome shotgun approach and using plasmid or fosmid libraries as templates for dye-terminator sequencing (Sanger sequencing). In determining the chromosome sequences of *A. brassicae* and *A. palmae*, a combination of Sanger sequencing and next generation sequencing methods (pyrosequencing, 454 Life Sciences/Roche) was applied for the first time to this bacterial family [7].

Both taxa show characteristic gene losses. In comparison to acholeplasmas, phytoplasmas lack the F_1_F_O_ ATPase synthetase complex, the cell division protein FtsZ, a wider variety of ABC transporters, the Rnf complex and the membrane protein SecG of the *Sec*-dependent secretion system. Moreover, acholeplasmas possess a rich repertoire of enzymes involved in carbohydrate metabolism, fatty acids, isoprenoids and partial amino acid metabolism [7]. Because these findings were inferred from the analyses of three acholeplasma and five phytoplasma genome sequences, it remains unclear as to what extent these differences between the two genera can be truly generalised or if the other acholeplasmas might share some of these features of their genetic repertoire with the phytoplasmas. Therefore, we determined the complete genome of *A. oculi* strain 19L by applying two different strategies based on sequencing by synthesis (SBS, Illumina) and, in a second approach, single molecule real time (SMRT, PacBio) sequencing. The subsequent analyses highlight the efficiency of current sequencing technologies and provide remarkable insights into the evolution of *Acholeplasmataceae*.

## Results

### Comparison of assemblies derived from SBS and SMRT sequencing

**Figure 1:**
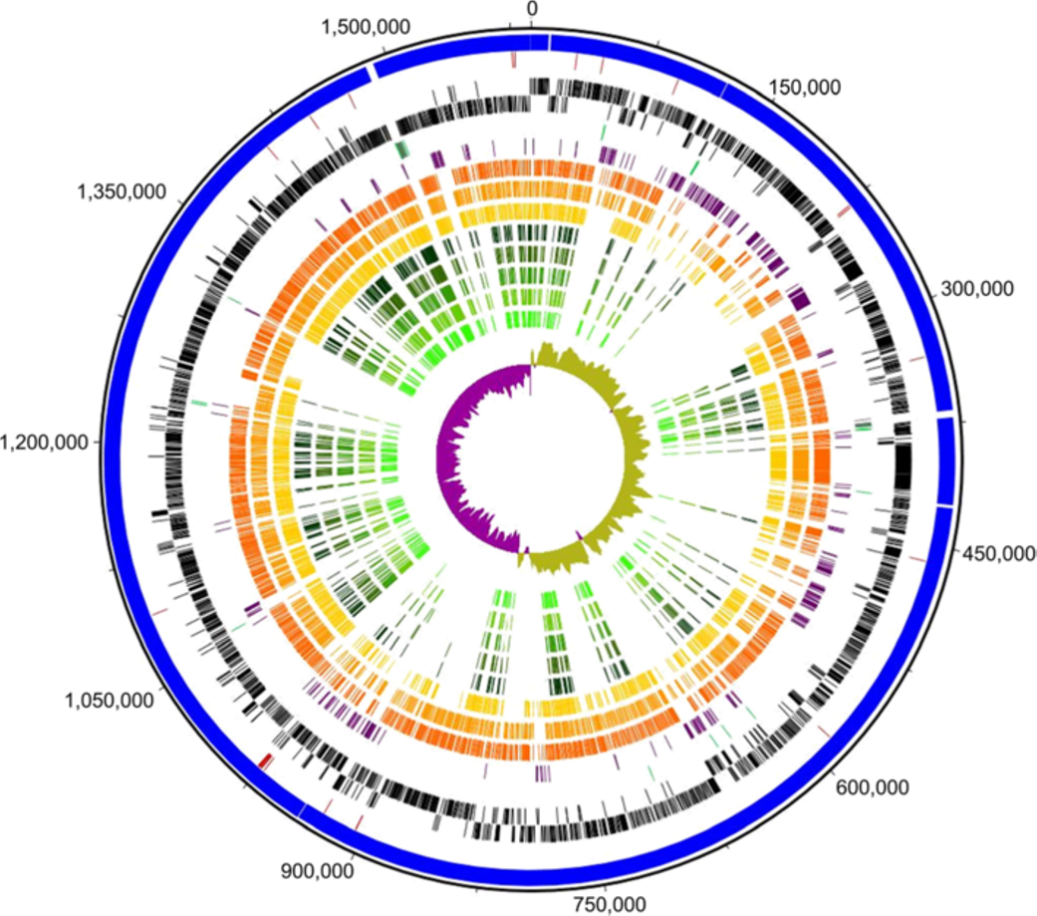
**Genome circle of** ***Acholeplasma oculi*** **strain 19L and a summary of the comparative analyses.** Circular patterns (from outside to inside): 1 (outer circle), scale in base pairs of the chromosome; 2 (blue), six contigs obtained from the initial SBS paired-end read assembly; 3 (red), 28 differences identified by comparing the results of the Illumina and PacBio sequencing results; 4 (black), predicted proteins encoded on the forward and reverse strands; 5 (green) tRNA genes and (grey) rRNA operons; 6 (violet), predicted unique proteins for *A. oculi* in comparison to other *Acholeplasmataceae* species; 7 (orange), orthologous proteins of *A. oculi* and *A. laidlawii*; 8 (light orange), orthologous proteins of *A. oculi* and *A. brassicae*; 9 (yellow), orthologous proteins of *A. oculi* and *A. palmae*; 10-14 (green), orthologous proteins of *A. oculi* and ‘*Ca*. P. asteris’ strain OY-M, AY-WB, ‘*Ca*. P. australiense’ strain rp-A, NZSb11 or ‘*Ca*. P. mali’ strain AT, respectively; 15 (olive and purple), cumulative G + C skew.

The SBS and SMRT approaches enabled the efficient reconstruction of the complete genome sequence in independent experiments. SBS sequencing provided 1,095,574 paired-end quality passed reads with an average length of 101 nt (total read length of 110,652,974 nt). *De novo* assembly of the SBS-derived reads alone led to the incorporation of 964,613 reads (88%) into six large contigs (513260 bp, 244477 bp, 109253 bp, 547590 bp, 106516 bp and 52461 bp in size), showing a 64-fold read coverage on average (Table 1, Figure 1) and reaching a total contig length of 1,573,557 bp. In turn, the mapping of SBS reads on the finished genome sequence revealed no uncovered regions. Gap regions derived from the SBS *de novo* assembly cover repetitive sequences of high similarity. In detail, two gaps (4,748 bp and 4,898 bp in size) include the rRNA operon regions (99% sequence identity), two gaps (1,661 bp each) include two transposases (100% sequence identity), one gap (186 bp) borders *Acholeplasma* phage L2 (>92% sequence identity) and the smallest gap (176 bp) is located close to a heavy metal translocating P-type ATPase (92% sequence identity). Gaps derived from the assembly of SBS reads were closed by primer-walking (Sanger sequencing), resulting in a complete circular chromosomal sequence.

**Table 1:** **Results obtained by the** ***de novo*** **assembly of paired-end SBS reads**

Name	Consensus length [bp]	Total read count	Average coverage
contig1	513,260	287,207	56.16
contig2	244,477	159,855	65.63
contig3	109,253	78,395	72.03
contig4	547,590	331,384	60.74
contig5	106,516	77,108	72.65
contig6	52,461	30,664	58.65
total	1,573,557	964,613	64.31

The SMRT sequencing approach provided 42,300 SMRT reads with a mean read length of 6,747 nt (total read length of 285,414,973 nt). A total of 38,875 reads enabled the gapless reconstruction of the circular chromosome at a size of 1,587,116 bp. A consensus concordance of 99.9991% and 144.1-fold average sequence coverage were reached. Around 8% of the SMRT-derived reads were rejected due to insufficient quality or incomplete read processing during the assembly.

Apart from the 14 kb size difference in the total contig length obtained by the two methods, it is remarkable that only 28 positions differ, concerning 31 bases in total (Table 2). Amplifying these positions, and re-sequencing by Sanger, led to the unambiguous assignment of 22 positions. In addition, the results indicated the presence of six polymorphic positions. Thus, both approaches enabled the efficient reconstruction of the complete chromosome sequence. The final sequence is in accordance with the results obtained through the Sanger sequencing of PCR products covering the ambiguous regions. Consequently, only polymorphisms occurring at high frequency in one PCR template were detected as double peaks in the chromatograms. We conclude that the majority of differences indicate that polymorphisms were already present in the original cell population. Furthermore, one of the substitutions results in a non-synonymous exchange (Aocu_13520/ *rpsE*) within the 30S ribosomal protein S5, while three substitutions result in synonymous base exchanges (Aocu_08380, Aocu_10040/ *mutS*, Aocu_14610) and two positions are located in intragenic regions. A high incidence of differences (14 nucleotides, Table 2) was identified within a putative phage-associated region (position 972,742-977,480), which may undergo rapid degradation and comprises an integrase (Aocu_08840) and fragments of a restriction-modification system (truncated restriction endonuclease, Aocu_08860; truncated restriction endonuclease, Aocu_08870 and *hsdM*, Aocu_08880).

**Table 2:** **Evaluation of differences by PCR and Sanger sequencing**

Primer	Sequence 5′- 3′	No. of differences	Position of the conflict	SMRT	SBS	Sanger
Aocu1FS1	ACGCAATTTTGAATGCGAGTC	1	29,164	A	G	G
Aocu1RS1	AAGCGCCACCCATCTTTACA					
Aocu2FS2	TCCAAGATCAACCGTTGGAACA	1	45,841	C	T	T
Aocu2RS2	TCTTTGTGCCTCACCACCTG					
Aocu3FS3	GGCAGTTGGTACAAGAGCGA	1	94,414	A	C	C
Aocu3RS3	AAATTCCGGTGGTGGTACGG					
Aocu45FS4	ACAGTTGATGGAAGCTATGAAGG	2	227,147	A	C	C
Aocu45RS4	ACGGTTGTTGGGAATCATGG		228,403	A	G	G
Aocu6FD5	ACTGCAGCTAATCCAACGGA	1	332,224/5	G	-	-
Aocu6RD5	TTCAAGTGTTCCACGTCGGT					
Aocu7FS6	TCAGCATCCGGTTATGCTCC	1	460,047	G	A	A
Aocu7RS6	GTTGGATGCCACTCGAAGGA					
Aocu8FS7	TCTTGTCTTGACCACCCCAA	1	584,955	A	G	G
Aocu8RS7	CAGCAAGTGTTTGACTCGCA					
Aocu910FS8	AAATCAGTTGCTGCATTAAGAGGT	2	904,938	G	C	S*
Aocu910RS8	ACTGGGAGTATCGATTGCAGG		904,961	C	A	M*
Aocu11FS9	TTAGATAGTGCGGCAAGGGG	1	927,019	G	A	R*
Aocu11RS9	AACGCACCGAATCATTTCGC					
Aocu12-FDI10	GTCGATGCGCAAGCATAACC	4	972,742/3	T	-	-
Aocu12-RDI10	TCTAGGAGGAACACCATCACG		974,678/9	–	AG	AG
			974,999/975,000	–	CT	CT
			975,166/7	–	AG	AG
Aocu16-22FDI11	TGCTAGCTGACCTTATGGGAC	7	976,093/4	T	-	-
Aocu16-22RDI11	GACGTTTAGGCGAAGTAGTCG		976,419	-	A	A
			976,451/2	T	-	-
			976,520/1	G	-	-
			976,525	-	T	T
			976,634/5	T	-	-
			977,480	-	T	T
Aocu23FS12	GCAATACGACCAACCAAGCG	1	1,091,760	T	C	Y*
Aocu23RS12	AATGCGCCAATTCCAAAACG					
Aocu24FS13	GACCAACGTTTCTCGCATGG	1	1,410,995	A	G	G
Aocu24RS13	TGACCACTTAGGTAATCGTCGT					
Aocu25FS14	AACTTGGTCCATGTGCCTCT	1	1,443,728	A	G	G
Aocu25RS14	ATGAGGCTACCATTACCCGC					
Aocu26FS15	ACCTCGATTGTTCCACCAGC	1	1,471,015	C	T	Y*
Aocu26RS15	AAGTGCTCGCTTACGTCTGG					
Aocu278FSI16	TTGCTTGGTTAGCTCCTCCC	2	1,576,028	T	A	W*
Aocu278RSI16	TAGGTGTGCGTCCTGAAGGT		1,577,778	-	T	T

Sixteen primer pairs were designed for the PCR and Sanger sequencing of 28 different regions resulting from the SMRT (PacBio) and SBS (Illumina) approaches. Primer pairs, numbers of identified differences, positions in the submitted sequence, SMRT- and SBS-determined sequences in any particular position are provided in addition to the Sanger sequencing results derived from the PCR product. Ambiguous results obtained by Sanger sequencing (*) were interpreted from the sequencing chromatograms.

In summary, Sanger sequencing confirmed the SBS-derived sequences for 22 out of 28 differences. The deviating SMRT data at these positions may indicate errors or rare sequence variations within the final chromosome sequence of 1,587,120 bp. Polymorphic sites at six sequence positions of the chromosome are supported by SMRT assembly, deviating SBS reads and Sanger sequencing.

### Benchmarks of the genome of *A. oculi*and its comparison to other *Acholeplasmataceae*

**Figure 2:**
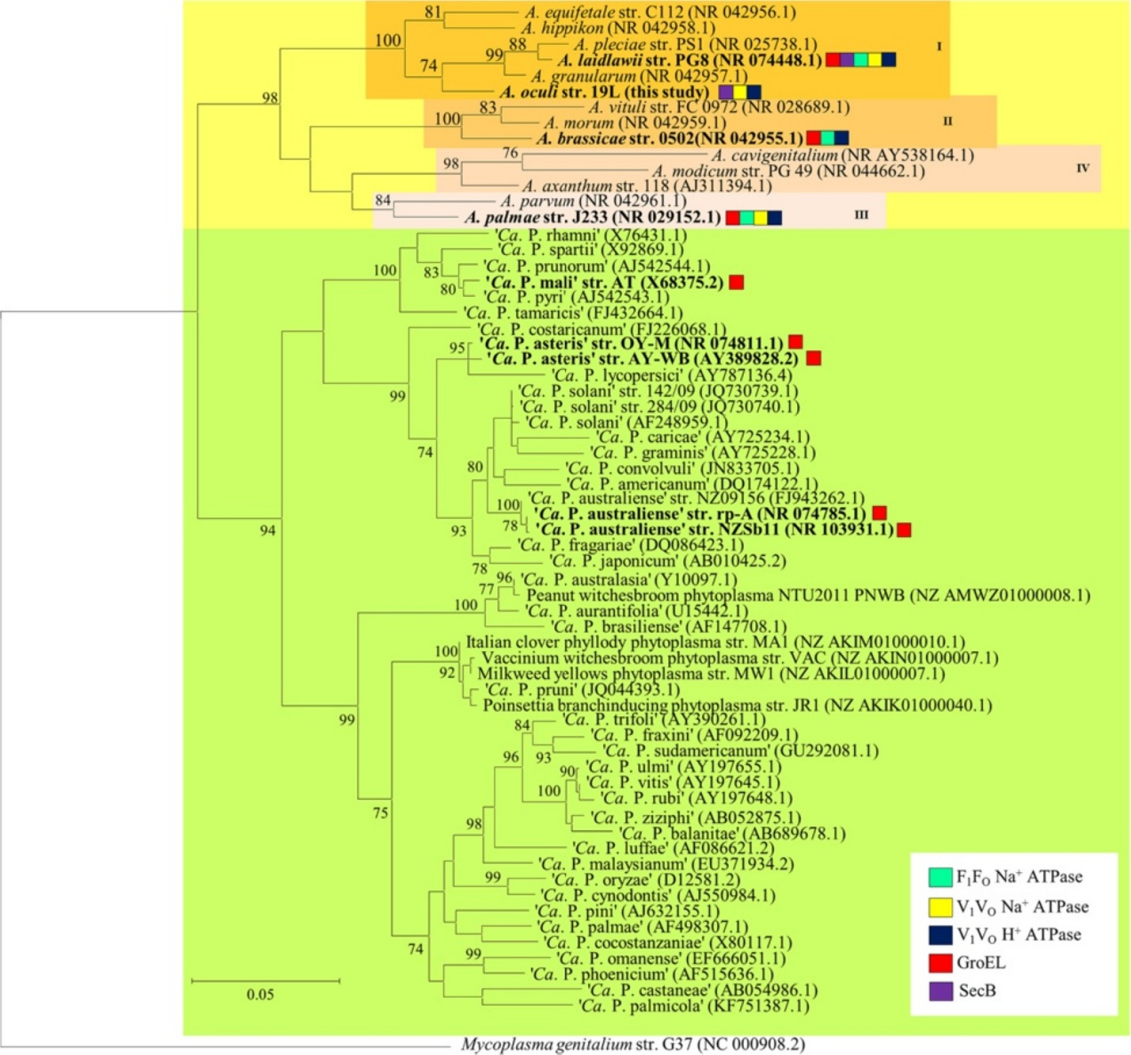
**Phylogenetic tree based on 16S** ***rRNA*** **gene sequences of acholeplasmas (orange) and phytoplasmas (green).** The tree was calculated by employing the maximum likelihood algorithm and bootstrap calculation for 1,000 replicates (only values of at least 70% are shown). The bar indicates 0.05 substitutions per nucleotide. The accession numbers are given in parentheses. *Mycoplasma genitalium* strain G37 is set as an out-group. Species with complete chromosomes available are shown in bold. Roman numerals are given according to acholeplasma clades [14]. The coloured boxes indicate that gene encoding F_1_F_0_ Na^+^ ATP synthase (light blue), V_1_V_O_ Na^+^ ATP synthase (yellow), V_1_V_O_ H^+^ ATP synthase (dark blue), GroEL (red) or SecB (violet) are present (limited to complete genome sequences).

**Figure 3:**
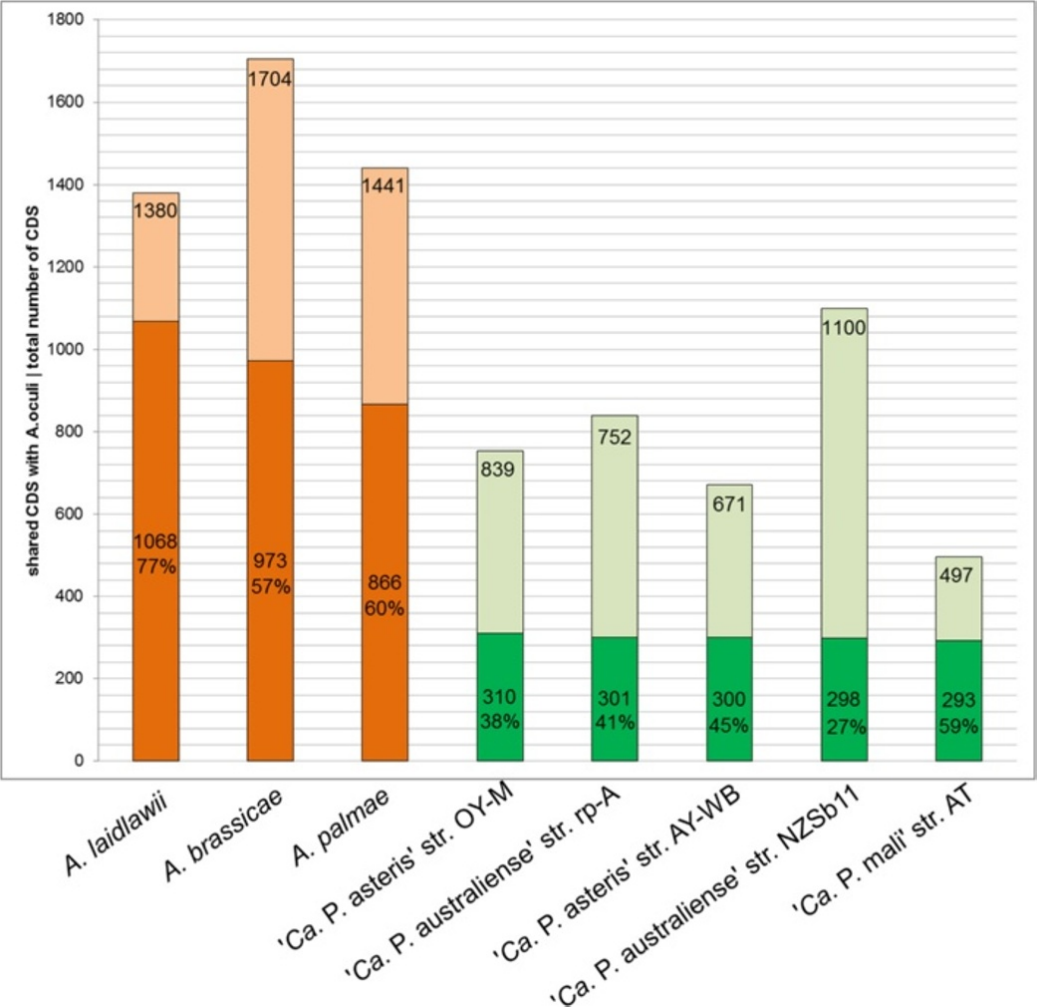
**Bar chart of shared protein content.** The total number of annotated protein coding genes (lighter part) for each complete genome sequence is given, together with the number and percentage of orthologous proteins with *A. oculi* in the lower part of each bar (darker part). PanOCT was used to predict orthologous proteins.

**Figure 4:**
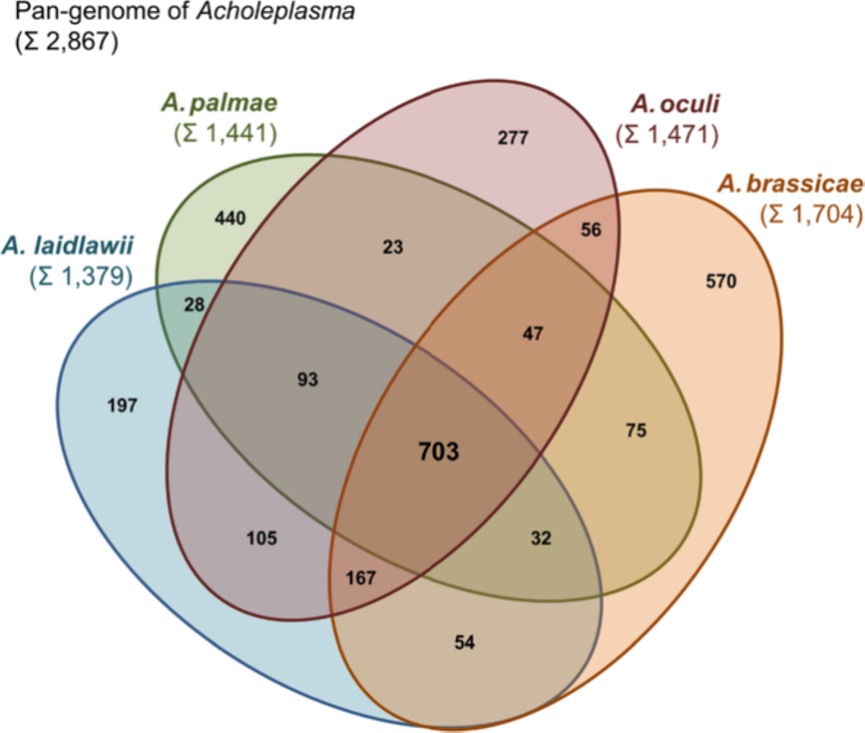
**Four-set Venn diagram of the pan-genome of the genus** ***Acholeplasma*** **based on the prediction of orthologous proteins by PanOCT.** Each ellipse shows in sum the total number of coding sequences of one *Acholeplasma* species. Intersections indicate predicted shared content.

The finished consensus sequence of *A. oculi* strain 19L consists of 1,587,120 bp encoding two rRNA operons, 34 tRNA genes and 1,471 predicted protein coding genes (Table 3). *A. laidlawii* strain PG-8A is the closest known relative of *A. oculi* strain 19L, which is supported by the construction of the phylogenetic tree (Figure 2). This close relationship is also reflected by the prediction of 1,068 shared proteins (77%) compared to 866 (60%) and 973 (57%) proteins shared with *A. brassicae* strain O502 and *A. palmae* strain J233 (Figure 3). The predicted core of the four *Acholeplasma* spp. consists of 703 proteins and the calculated pan-genome comprises 2,867 proteins in total (Figure 4). The highest number of unique proteins (570) possesses *A. brassicae*, which also exhibits the largest genome in this family (1,877,792 bp, 1,704 protein coding genes, Table 3).

**Table 3:** **Overview of the currently completely determined** ***Acholeplasmataceae*** **genomes**

Genus	***Acholeplasma***				‘***Candidatus***Phytoplasma’				
Species	***oculi***	***laidlawii***	***brassicae***	***palmae***	mali	australiense		asteris	
Strain	19L	PG-8	0502	J233	AT	rp-A	NZSb11	OY-M	AY-WB
**Chromosome organization**	**circular**	circular	circular	circular	linear	circular	circular	circular	circular
**Size [bp]**	**1,587,120**	1,496,992	1,877,792	1,554,229	601,943	879,959	959,779	853,092	706,569
**G + C [%]**	**31.29**	31.93	35.77	29,98	21.39	27.42	27.19	27.76	26.89
**CDS**									
Number (pseudogenes)	**1,471**	1,380	1,704 (14)	1,441 (2)	497 (15)	839 (155)	1100	752	671
G + C [%]^1^	**31.49**	32.23	36.15	29.20	22.60	28.72	28.47	29.09	28.54
Average length^1^	**992**	984	1,003	979	957	825	674	829	776
Coding percentage^1^ [%]	**92.0**	90.7	90.3	90.6	76.6	64.1	77.3	73.1	73.7
**rRNA**									
Number	**6**	6	14	6	6	6	6	6	6
G + C [%]	**49.27**	48.57	49.31	48.64	44.32	46.37	46.61	45.95	46.14
**tRNA**									
Number	**34**	34	45	35	32	35	35	32	31
G + C [%]	**56.87**	56.97	56.12	56.56	52.41	54.11	54.11	53.61	53.6
**plasmdis**									
Number	**-**	-	-	-	-	1	1	2	4
	**LK**	CP	FO	FO	CU	AM	CP	AP	CP
**Acc. no.**	**028559**	000896	681348	681347	469464	422018	002548	006628	000061
	***this study***	[8]	[7]	[7]	[13]	[9]	[10]	[11]	[12]

Bold letters highlight results obtained in this study. ^1^excluding pseudogenes.

**Figure 5:**
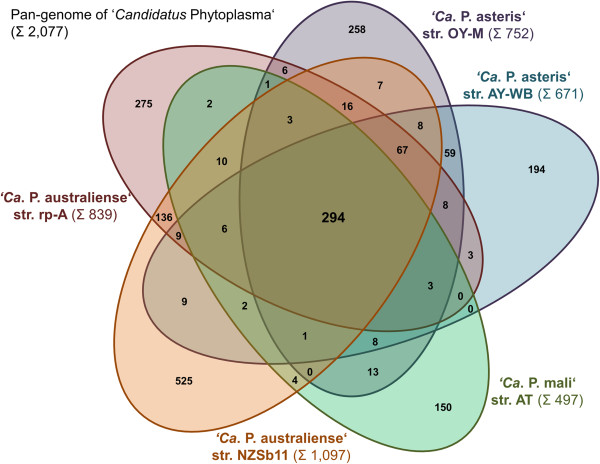
**Five-set Venn diagram of the pan-genome of the genus ‘** ***Ca.*** **Phytoplasma’ based on the prediction of orthologous proteins by PanOCT.** Each ellipse shows in sum the total number of coding sequences of one strain. Intersections indicate predicted shared content.

**Figure 6:**
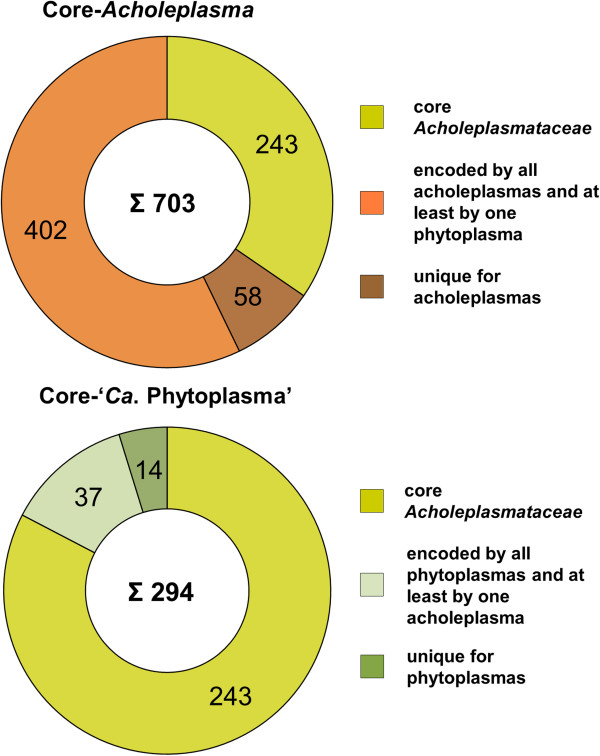
**Composition of the** ***Acholeplasma*** **and ‘** ***Ca*** **. Phytoplasma’ core-genomes as predicted by PanOCT.** The total number of proteins inferred from the respective core genome is given in the middle (uncoloured part).

Only a basic set of proteins is shared between *A. oculi* and the five complete phytoplasma genomes. The 293 to 310 predicted shared proteins (27% to 59%) are consistent with previously calculated numbers for other acholeplasmas [7, 8] (Figure 3). The second highest number of unique proteins (440) is predicted for *A. palmae* (Figure 4), which is the closest known relative of the phytoplasmas [7] (Figure 2) and is also supported by the highest number of predicted shared proteins with the five completely sequenced phytoplasmas (Additional file 1). In second position among the acholeplasmas, *A. oculi* shares many of its proteins with the phytoplasmas, supported by its phylogenetic assignment and the received PanOCT results. This analysis is also supported by *A. oculi* and *A. laidlawii*, which share the highest number of proteins amongst the acholeplasmas (Figure 3). The phytoplasmas’ genome reduction process is reflected by the low number of 294 proteins assigned to the shared core within the pan-genome (2,077 proteins in total; Figure 5). Phytoplasma genomes are characterised by extensive gene losses, transposon-mediated gene duplication [12] and horizontal gene-integration events [15]. Comparing the pan-genomes of acholeplasmas and phytoplasmas, Venn analysis highlights basic differences in the overall gene content. Complete *Acholeplasma* and ‘*Ca*. Phytoplasma’ genomes collectively encode 402 and 14 predicted unique proteins, respectively (Figure 6, Additional file 2). The 14 unique genes, which are common to the genus ‘*Ca*. Phytoplasma’, encode nine hypothetical proteins and five proteins with known functions. Two of the hypothetical proteins contain a sequence-variable mosaic (SVM) motif [16] and comprise SAP05 (AYWB_032), which is described as a putative effector protein [17] inducing the formation of smooth young rosette leaves that lack serrations along the leaf margin [18], and SAP30 (AYWB_402), which is similar to SAP11 containing an eukaryotic nuclear localisation signal [19, 20]. This group of unique genes also includes two phytoplasma proteins involved in a suggested alternative pathway in the carbohydrate metabolism of phytoplasmas [7, 13, 21]. The malate/Na + symporter (MleP) provides a carbon source which undergoes oxidative decarboxylation by malate dehydrogenase (SfcA), thereby providing pyruvate processed by the dehydrogenase multienzyme complex and providing acetyl coenzyme A. The phosphotransacetylation of acetyl-CoA performed by the PduL-like protein provides acetyl-phosphate, which is processed via acetate kinase (AckA) and results in the formation of ATP and acetate. *A. oculi* does not encode MleP, SfcA and the phosphate acetyltransferase (Pta), which is common in mycoplasmas, though it is suggested that PduL fulfills this function in *Acholeplasmataceae* [7, 21] including *A. oculi*. However, the alternative energy-yielding pathway of phytoplasmas utilising malate is clearly not encoded in the analysed acholeplasma genomes.

Furthermore, the PanOCT analysis predicted that phytoplasmas encode unique AAA+ ATPase, thymidylate kinase and a DNA-dependent RNA polymerase sigma 70 factor RpoD (IPR013325, IPR014284, IPR007627, Additional file 2). RpoD exhibits only insignificant BlastP hits to acholeplasmas’ sigma factors (minimal e-value 9e-08, score 47), and no ortholog was predicted via PanOCT. The existence of a phytoplasma-specific sigma factor points towards some peculiarities in their regulatory system. The other two deduced proteins showed similarities in BlastP analysis to some acholeplasma proteins, albeit they differed in small domain structures. For instance, the AAA+ ATPase of phytoplasmas gave a hit to the ATP-dependent zinc metalloprotease FtsH, which also contains the AAA+ domain structure, and the thymidylate kinases of acholeplasmas showed an additional conserved site (predicted by the PROSITE database search, http://prosite.expasy.org/) – contrary to the thymidylate kinases of phytoplasmas. The overall high number of 402 unique proteins for the four acholeplasmas is interpreted with respect to the diverse environments colonised by acholeplasmas.

### Particularities of *A. oculi*strain 19L

A high percentage (55% – 148) of the 271 proteins predicted to be ‘unique’ for *A. oculi* in the *Acholeplasmataceae* are annotated as hypothetical proteins (Figure 1). This set of unique proteins contains phage-related proteins similar to the *Acholeplasma* phage L2 [GenBank: L13696.1] [22], including the proteins L2_7, L2_9, L2_11 and L2_12, and which are organised in clusters at three different chromosome regions (118,599-126,732 Aocu_01170-01290; 650,878-659,350 Aocu_05790-5880; 1,062,230-1,068,906 Aocu_09710-9810). In addition, two recombinases (Aocu_08890, Aocu_13990), two integrases (Aocu_05790, Aocu_08840) and one resolvase (Aocu_14000) belong to the list of unique proteins. Besides phage-assigned elements, six transposases were identified, including a mutator type (Aocu_01380), IS3/IS911 family protein (Aocu_01570), IS200-like (Aocu_03680), IS204/IS1001/IS1096/IS1165 (Aocu_04230/50) and other transposon-related elements (insertion element subunit, Aocu_03690). Furthermore, a putative complex transposon is associated with a region carrying a high number of unique proteins (134,519-157,461, Aocu_01380-01570). Beside other proteins, it encodes two oligopeptide ABC transporter components (periplasmatic component *oppA* and ATP-binding protein *oppD*) and six glycosyl hydrolase family proteins and a periplasmatic binding protein/LacI transcriptional regulator. A second candidate for a complex transposon (423,889-490,302 Aocu_03680-4250) encodes a UDP-galactopyranose mutase (Glf, Aocu_04190) in addition to another Glf (Aocu_04670) outside the proposed complex transposon. These genes set *A. oculi* apart from other acholeplasmas. The UDP-galactopyranose mutase is involved in the conversion of UDP-galactopyranose (UDP-GALP) into UDP-galactofuranose (UDP-GALF) (IPR004379), which is the precursor to D-galactofuranose and is often found in the lipopolysaccharide O antigens of several Gram-negative bacteria [23].

*A. oculi* is also separated from the other three acholeplasmas by the presence of a putative manganese efflux pump (MntP, Aocu_03470), thereby enabling the exportation of manganese ions, which are toxic in higher amounts. The functional relevance of MntP for manganese homoeostasis has been demonstrated for *E. coli* [24]. The direct comparison of the *A. oculi* and the *E. coli* MntP protein shows 31% identical and 56% similar residues. In addition, *A. oculi* encodes a cadmium resistance transporter (CadD, Aocu_08600) and one amidohydrolase (AmhX, Aocu_08940). In *Bacillus subtilis*, AmhX enables the cleavage of the amide bond between non-active conjugated amino acids and may mobilise indole-3-acetic acid (IAA) from inactive storage forms in plants besides several other functions [25] (IPR017439 [26]). *A. oculi* was also detected on plant surfaces [27]. Therefore, one may speculate whether *A. oculi* can stimulate the growth of colonised plant tissue. Hints for such a manipulation of the IAA metabolism of plants have also been obtained for *A. palmae* and *A. brassicae* encoding a putative auxin efflux carrier protein [7], though no experimental studies are available.

*A. oculi* is separated from the other *Acholeplasma* spp. by encoding several additional transcriptional regulators such as *ubiC* (Aocu_00680), *gntR* (Aocu_00690), Cro/C1 family proteins (Aocu_01770, Aocu_05750, Aocu_08910 and Aocu_13020) and TetR family proteins (Aocu_14450) not assigned to other Cro/C1-type or TetR family proteins in this family. In total, *A. oculi* encodes 13 Cro/C1 family proteins, nine of which are shared, and four TetR family proteins, one of which is shared by the other acholeplasmas.

Furthermore, *A. oculi* is separated from other *Acholeplasmataceae* by encoding the GDP-D-glycero-α-D-manno-heptose biosynthesis pathway providing D-glycero-D-manno-heptose (HddA, GmhA, HddC, GmhB; Aocu_04590-620). This is a precursor of the inner core lipopolysaccharide [28]. These proteins are similar to those found in the pathway that was reconstructed for the Gram-positive bacteria *Aneurinibacillus thermoaerophilus* strain DSM 10155 (member of *Bacillus*/*Clostridium* group) [28]. For acholeplasmas, there is only one report by Mayberry *et al*. [29] that *A. modicum* contains heptose among the glycolipids.

Moreover, *A. oculi* encodes two additional proteins, thus playing a role in the biosynthesis of the amino acid methionine. MetW (Aocu_08790) synthesises methionine from homoserine (IPR010743 [30]), which provides an additional pathway to produce methionine needed in the initiation of translation. The diaminopimelate epimerase (DapF, Aocu_08990) belongs to the aspartate pathway (IPR001653), from which the four amino acids lysine, threonine, methionine and isoleucine can be synthesised.

**Figure 7:**
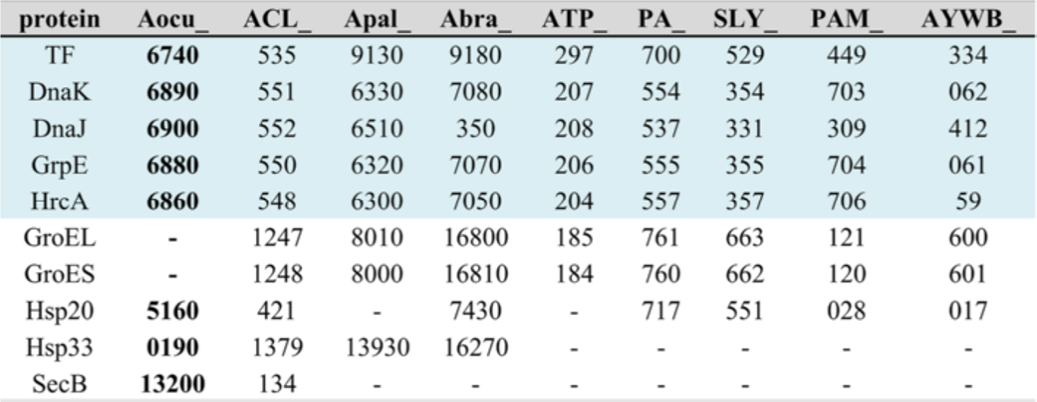
**Locus tags of the encoded chaperons and heat shock proteins within the** ***Acholeplasmataceae.*** Abbreviations: *Acholeplasma oculi,* Aocu; *A. laidlawii,* ACL; *A. palmae*, Apal; *A. brassicae*, Abra; ‘*Candidatus* P. mali’ strain AT, ATP; ‘*Ca*. P. australiense’ strain rp-A, PA; ‘*Ca*. P. australiense’ strain NZSb11, SLY; ‘*Ca*. P. asteris’ strain OY-M, PAM; ‘*Ca*. P. asteris’ strain AY-WB, AYWB. Shared proteins are highlighted in blue.

All species of the *Acholeplasmataceae* encode a protein core for the *Sec*-dependent secretion system (Ffh, FtsY, SecA, SecE, SecY and YidC), whereas the four analysed *Acholeplasma* spp. additionally encode the membrane protein SecG. The chaperone SecB, which is only encoded in *A. laidlawii* and *A. oculi*, binds the precursor protein and directs it to the SecA protein. The function of SecB can also be fulfilled by the proteins DnaK and DnaJ [31], which are encoded in all genome sequences of the family, or by GroEL and GroES [32]. *A. oculi* lacks the common chaperone GroEL/ES (Figure 7), consistent with conclusions drawn from the draft sequences of phytoplasma strains [33] and the analyses of other species in the *Mollicutes* that these genes are not essential [34]. The complete genome sequences of the *Acholeplasmataceae* encode the trigger factor (TF), *dnaK*, *dnaJ*, *grpE* and *hrcA*. Other heat shock proteins, such as Hsp20, were not identified in *A. palmae* and ‘*Ca*. P. mali’. Hsp33 is only identified in the acholeplasmas.

**Figure 8:**
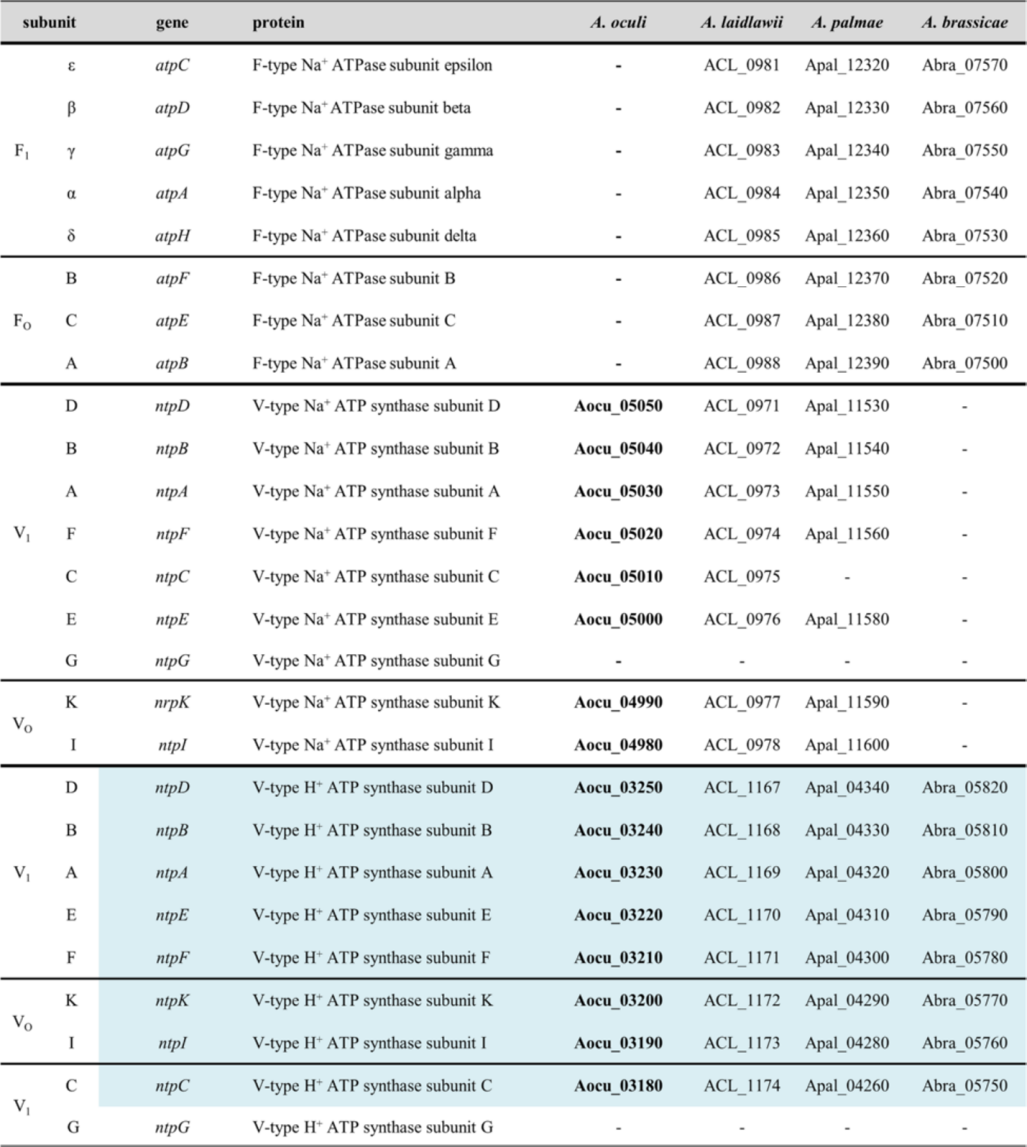
**An overview of the subunits of the F- and V-type ATPases encoded by acholeplasma genomes.** The subunit order follows location within the chromosomes. Genes shared by all of the four acholeplasmas are highlighted in blue. Numbers indicate locus tags corresponding to the deduced proteins.

**Figure 9:**
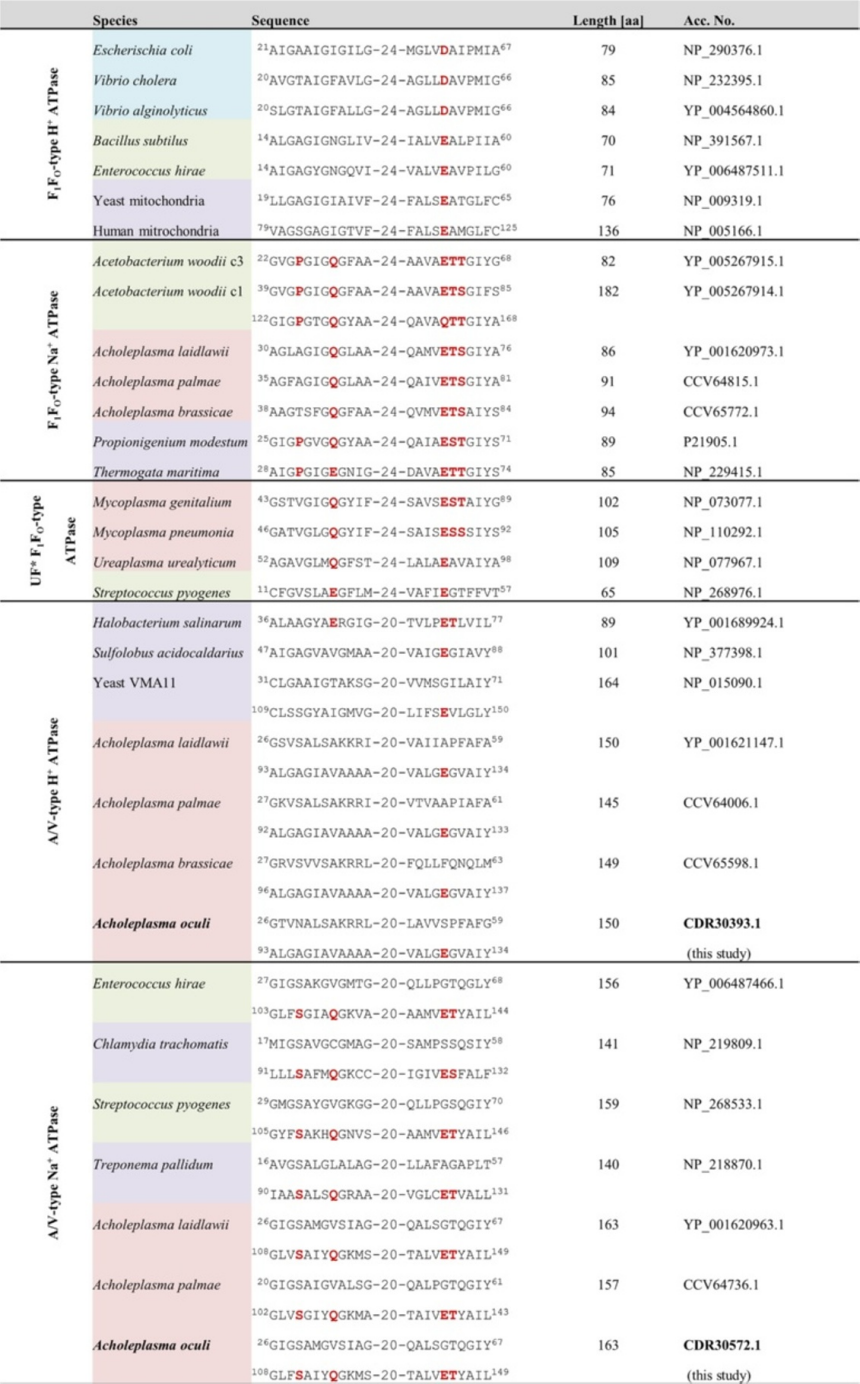
**Alignment made by partial protein sequences of the F-type ATPase subunit c (AtpE) and the V-type ATPase subunit K (NtpK).** Assignments were made according to Dzioba *et al*. [36]. Species are highlighted regarding their phylum assignment to *Tenericutes* (red), *Firmicutes* (green) or others (violet). Superscripted numbers indicate position in protein sequence. *UF = unknown function.

Beside GroEL/ES, *A. oculi* lacks the complete gene set encoding the F_1_F_O_-type Na^+^ ATPase, which was identified in *A. laidlawii*, *A. brassicae* and *A. palmae* (Figures 8 and 9). Therefore, *A. oculi*, *A. laidlawii* and *A. palmae* encode one V-type Na^+^ ATPase. *A. palmae* differs in gene content by encoding no *atpC* subunit for this ATPase. In addition, all genes encoding the V_1_V_O_ H^+^ ATPase are present in all four acholeplasma strains. Summing up, each acholeplasma species possesses at least one full operon which encodes at least either one H^+^ or one Na^+^ ATPase system. The NtpG subunit, namely the rotated central stalk next to NtpD and NtpC [35], is missing in all species. In contrast to the acholeplasmas, the F- and V-type ATPases were not identified in phytoplasmas.

## Discussion

F_1_F_O_ ATPases and V_1_V_O_ ATPases are membrane complexes which function either as H^+^- or Na^+^-translocators [37] (Figure 9). The F_1_F_O_ ATPase consists of two units – the integral membrane protein F_O_ (*atpBEF*) acting as a proton channel and the peripheral catalytic stalk F_1_ (*atpHAGDC*). The V_1_V_O_ ATPase is built by the integral membrane protein V_O_ (*ntpIK*) and the peripheral catalytic stalk V_1_ (*ntpECFABD*) [38]. The difference between both transporters is that the V-type ATPase only works in one direction by hydrolysing ATP to produce either a proton or a sodium motive force, while additionally the F-type ATPase is able to act in the other direction by allowing the regulation of the cellular ion pool using the proton motive force, which leads to ATP generation [39].

Following the sequence-based prediction of Dzioba *et al*. [36], the classification of the ion translocating profile can be inferred from the alignment of the protein sequences of the subunits AtpE (F-type ATPase) and NtpK (V-type ATPase). Certain conserved binding motifs are represented by the amino acids at specific positions, in order to specify an H^+^- or a Na^+^-translocation (Figure 9). As a result, one F_1_F_O_-type Na^+^ ATPase is suggested to be encoded by all acholeplasmas except for *A. oculi*, and one V-type Na^+^ ATPase is predicted for all acholeplasmas except for *A. brassicae*. It remains unclear as to whether the V-type Na^+^ ATPase of *A. palmae* is working despite the lack of an *atpC* subunit, although this species additionally encodes the F_1_F_O_-type Na^+^ ATPase. Moreover, protein sequence alignment leads to the conclusion that all acholeplasmas encode one V-type H^+^ ATPase. Deductively, all acholeplasmas encode at least one Na^+^ and one H^+^ translocator. This finding stands in accordance with the evidence that *Acholeplasma laidlawii* strain B possesses a (Na^+^-Mg^2+^)-ATPase which is capable of actively extruding sodium ions against the concentration gradient [40]. This previously described, but not genetically characterised, cation pump was linked to the characteristically low intracellular sodium level of these bacteria.

Ultimately, the loss of the F_1_F_O_-type Na^+^ ATPase in *Acholeplasmataceae*, as is the case for *A. oculi*, may probably be compensated by the V-type Na^+^ ATPase. The loss of this genetic module in phytoplasmas remains unclear, but it might be interpreted in respect to the adaptation of phytoplasmas in the intracellular environment with constant osmotic conditions. The comparison of both V-type ATPase operons encoded by *A. oculi* highlights low sequence identities of the involved proteins (24% to 52%) and differences in protein lengths (Figure 9). This leads to the suggestion that the operons did not derive from a duplication event.

Besides F_1_F_O_ ATPase, the loss of *groEL/ES* is remarkable. Native protein folding is conducted by molecular chaperones such as GroEL/ES (Hsp60), DnaK (Hsp70), DnaJ, GrpE, SecB and other heat-shock proteins (Hsp) [41]. GroEL complexes (800 kDa) consisting of two stacked heptameric rings exhibit ATPase activity [42]. The smaller GroES (10 kDa), together with ATP, binds to GroEL and forms the GroEL/GroES complex. DnaK prevents off-pathway reactions or stabilises certain folding intermediates. DnaJ and GrpE act as co-helpers for DnaK [41]. GroEL/ES is probably replaced by the trigger factor (TF) and DnaK, which has already been shown by Kerner *et al*. [43] for *E. coli* or by the HrcA protein, which is commonly found as a part of the heat-shock regulation of bacteria [44]. TF/DnaK and HrcA are encoded in all analysed species of the *Acholeplasmataceae* (Figure 7). Several *Mollicutes* are known to have lost *groEL* and *groES*, such as *Mesoplasma florum*, *Mycoplasma hyopneumoniae*, *Ureaplasma parvum serovar* 3, *Ureaplasma urealyticum*, *Mycoplasma mobile* and some further *Mycoplasma* spp. [44]. It is likely that there are even more *Mollicutes* lacking these proteins. Saccardo *et al*. [33] suggested, based on draft sequences, that there are four phytoplasma strains of the 16SrIII group that probably lack GroEL/ES. The possibility that this genetic feature can be lost within the *Mollicutes* is supported by experiments with transposon mutagenesis, showing that GroEL is not or only weakly regulated during heat shock for *M. genitalium* or *M. pneumonia* [45], thereby leading to the suggestion that this chaperone is not essential for *Mycoplasma* spp. in general and may represent an evolutionary remnant. Evolutionary loss could apparently be possible due to either the fact that GroEL is immunogenic, and therefore it would be advantageous to get rid of it by avoiding an immune response in mammals [44] – a benefit for *A. oculi* when infecting mammals – or alternatively *Mollicutes* possess small genomes which encode few proteins; consequently, they own fewer substrate proteins, which have to be correctly folded by GroEL.

## Conclusions

This study demonstrated the efficiency of the SMRT approach in the complete *de novo* determination of bacterial genomes. *A. oculi* encodes, like other *Acholeplasma* spp., rich genetic content in comparison to phytoplasmas. The relatively small core genome of phytoplasmas should be interpreted with respect to their intracellular parasitism and their corresponding poor metabolic repertoire. In contrast, acholeplasmas depend on a richer genetic repertoire due to their widespread distribution and colonisation of diverse micro-habitats. However, for the first time, the deduced protein content of *A. oculi* highlights that the loss of basic genetic elements, including the chaperone GroEL/ES and the F_1_F_O_-type Na^+^ ATPase system, took place in both genera of the *Acholeplasmataceae*. One could therefore speculate that the common V-type H^+^ ATPase system in acholeplasmas may regulate the cellular proton pool, and the V-type Na^+^ ATPase system may compensate for the lack of the F_1_F_O_-type Na^+^ ATPase. The loss of GroEL/ES is interpreted as being not extraordinary for *Mollicutes* and seems to have occurred several times within this class.

## Methods

### Cultivation

*A. oculi* strain 19L isolate was kindly provided by Jerry K. Davis (Purdue University School of Veterinary Medicine, West Lafayette, Ind., USA) from the strain collection of the International Organization for Mycoplasmology (IOM). Cells were cultivated in ATCC® Medium 1039 (http://www.atcc.org) supplemented with 0.2% polymyxin B (Roth, Karlsruhe, Germany) and 0.2% penicillin G (Merck, Darmstadt, Germany) at 28°C for about 14 days and collected by centrifugation (20 min, 10,000 rpm, 4°C). The DNA isolation of *A. oculi* strain 19L for SBS was performed with the DNeasy Blood & Tissue Kit (Qiagen, Hildesheim, Germany) and according to the manufacturer’s instruction. DNA isolation needed for preparing the PacBio 10-kb library high molecular weight genomic DNA was performed according to Moore *et al*. [46].

### Sequencing and assembly of SBS data

DNA-Seq libraries were prepared from fragmented DNA (COVARIS S2, Woburn, Massachusetts, USA) according to recommendations made by the supplier (TruSeq DNA sample preparation v2 guide, Illumina, San Diego, CA, USA). Libraries were quantified by fluorometry, immobilised and processed onto a flow cell with a cBot followed by sequencing by synthesis by applying TruSeq v3 chemistry on a HiSeq2500 (all components by Illumina).

The *de novo* assembly of the reads was performed in CLC Genomics Workbench 7.0 (http://www.clcbio.com). The assembly data was exported as a BAM file, indexed using SAMtools [47] and imported in Gap5 [48]. Gaps were closed by PCR and primer-walking by applying dye-terminator sequencing performed on an ABI 310 capillary sequencer (Life technologies, Carlsbad, CA, USA).

### Sequencing and assembly of SMRT data

A 10-kb library was prepared and processed as recommended by Pacific Biosciences (http://www.smrtcommunity.com/SampleNet/Sample-Prep). Library construction and subsequent sequencing were performed using the SMRTbell Template Preparation Reagent Kit 1.0, DNA/Polymerase binding kit P4-C2, MagBead Kit and DNA Sequencing Kit 2.0 (all components supplied by Pacific Biosciences, Menlo Park CA, USA.). The genome was sequenced using PacBio RS II technology (P4-C2 chemistry). Data collected on the PacBio RS II instrument were processed and filtered (SMRT analysis software, version 2.1). All experiments were conducted according to the manufacturers’ instructions on a single SMRT cell. Obtained data were analysed on the SMRT Portal V2.1.1 (http://www.pacb.com/devnet/) by applying the integrated Celera® Assembler. SMRT sequencing and SBS were performed by the Max Planck-Genome-centre Cologne, Germany (http://mpgc.mpipz.mpg.de/home/).

### Identification of sequencing differences comparing both sequencing methods

Rare sequencing differences were identified via BlastN (low complexity filter off, word size 7) [49] by applying the SMRT-derived genome sequence as a reference. In addition, SBS data were mapped onto the SMRT sequence in CLC Genomics Workbench 7.0, and Primer-BLAST [50] was used for designing oligonucleotide pairs, thus enabling the PCR amplification of conflict regions (Table 2). Sequences of PCR products were determined by applying dye-terminator sequencing.

### Annotation of the genome sequence

The *oriC* region was determined through the cumulative GC-skew calculation of the chromosome sequence in Artemis [51] and the determination of the DnaA-boxes [8]. The adjusted genome sequence was automatically annotated in RAST [52] and annotation was manually curated in Artemis by incorporating additional analyses obtained from the InterProScan database [53], RNAmmer [54] and tRNAscan-SE [55]. The annotated genome sequence of *A. oculi* strain 19L, including information on polymorphisms, was deposited [EMBL:LK028559]). Read data for SBS [EMBL:ERX463488] and SMRT [EMBL:ERX470328] were submitted to the European Nucleotide Archive (http://www.ebi.ac.uk/ena/).

### Prediction of orthologous proteins within the *Acholeplasmataceae*family

Orthologous proteins of *Acholeplasmataceae* were calculated by PanOCT [56] by applying the default parameters and protein data of *A. laidlawii* strain PG-8A [Genbank:CP000896.1] [8], *A. brassicae* strain O502 [GenBank:FO681348.1] [7], *A. palmae* strain J233 [GenBank:FO681347] [7], ‘*Ca*. P. australiense’ strain rp-A [GenBank:AM422018.1] [9] and NZSb11 [GenBank:CP002548.1] [10], ‘*Ca*. P. asteris’ strain OY-M [GenBank:AP006628.2] [11] and AY-WB [GenBank:CP000061.1] [12] and ‘*Ca*. P. mali’ strain AT [GenBank:CU469464.1] [13]. The results obtained by the software were also used for the prediction of the pan-, dispensable- and core-genome [57] of each genera and the family [58].

### Phylogenetic analysis of the *Acholeplasmataceae*

Alignment and the phylogenetic tree were calculated for 64 16S *rRNA* genes of the *Acholeplasmataceae* by using the maximum likelihood algorithm implemented in CLC Genomics Workbench 7.0 and by applying the parameter ‘very accurate’ for the alignment with a gap open cost of 10.0 and a gap extension cost of 1.0. The phylogenetic tree was constructed by using a 1,000 bootstrapped maximum likelihood algorithm, whereby the ‘UPGMA’ construction method and the ‘Jukes Cantor’ nucleotide substitution model were chosen. *Mycoplasma genitalium* strain G37 was added, in order to root the tree.

## Electronic supplementary material

Additional file 1: **Number of shared proteins for each species of the** ***Acholeplasmataceae*** **predicted by PanOCT.** Acholeplasmas are highlighted in orange and phytoplasmas in green. Predicted orthologs in phytoplasmas and acholeplasmas are highlighted in blue. The highest number of shared proteins between acholeplasmas and phytoplasmas is underlined. Abbreviations: *Acholeplasma oculi,* Aocu; *A. laidlawii,* Alai; *A. palmae*, Apal; *A. brassicae*, Abra; ‘*Candidatus* Phytoplasma mali’ strain AT, Pmal; ‘*Ca*. P. australiense’ strain rp-A, Paus; ‘*Ca*. P. australiense’ strain NZSb11, SLY; ‘*Ca*. P. asteris’ strain OY-M, OY-M; ‘*Ca*. P. asteris’ strain AY-WB, AY-WB. (DOCX 112 KB)

Additional file 2: **Listed orthologous proteins of the** ***Acholeplasmataceae*** **and ‘unique’ proteins of** ***Acholeplasma*** **and ‘** ***Candidatus*** **Phytoplasma’.** The pan analysis led to a core of 243 proteins for the family *Acholeplasmataceae*, to 402 ‘unique’ proteins for the genus *Acholeplasma* and to 14 ‘unique’ proteins for the genus ‘*Ca.* Phytoplasma’, respectively. Results were predicted by PanOCT analysis. The five completely sequenced phytoplasmas are highlighted in green, and the four completely sequenced acholeplasmas are highlighted in orange. (XLSX 102 KB)
