# Supplementary material for: Complete genome determination and analysis of Acholeplasma oculi strain 19L, highlighting the loss of basic genetic features in the Acholeplasmataceae
Source: BMC Genomics. 2014 Oct 24;15(1):931. doi: 10.1186/1471-2164-15-931 (PMC4221730; doi:10.1186/1471-2164-15-931)
Supplement: Supplementary file 1 — Additional file 1: Number of shared proteins for each species of the Acholeplasmataceae predicted by PanOCT. Acholeplasmas are highlighted in orange and phytoplasmas in green. Predicted orthologs in phytoplasmas and acholeplasmas are highlighted in blue. The highest number of shared proteins between acholeplasmas and phytoplasmas is underlined. Abbreviations: Acholeplasma oculi, Aocu; A. laidlawii, Alai; A. palmae, Apal; A. brassicae, Abra; ‘Candidatus Phytoplasma mali’ strain AT, Pmal; ‘Ca. P. australiense’ strain rp-A, Paus; ‘Ca. P. australiense’ strain NZSb11, SLY; ‘Ca. P. asteris’ strain OY-M, OY-M; ‘Ca. P. asteris’ strain AY-WB, AY-WB. (DOCX 112 KB) [file 12864_2014_6622_MOESM1_ESM.docx]

## Additional file 1. Number of shared proteins of each species of the *Acholeplasmataceae* predicted by PanOCT.

Acholeplasmas are highlighted in orange and phytoplasmas in green. Predicted orthologs in phytoplasmas and acholeplasmas are highlighted in blue. The highest number of shared proteins between acholeplasmas and phytoplasmas is underlined. Abbreviations: *A. oculi,* Aocu; *A. laidlawii,* ACL; *A. palmae*, Apal; *A. brassicae*, Abra; ‘*Ca*. P. mali’ strain AT, ATP; ‘*Ca*. P. australiense’ strain rp-A, PA; ‘*Ca*. P. australiense’ strain NZSb11, SLY; ‘*Ca*. P. asteris’ strain OY-M, PAM; ‘*Ca*. P. asteris’ strain AY-WB, AYWB.

|  | **Aocu** | **Alai** | **Abra** | **Apal** | **Pmal** | **Paus** | **SLY** | **OY-M** | **AY-WB** |
| --- | --- | --- | --- | --- | --- | --- | --- | --- | --- |
| **Aocu** |  | **1.068** | **973** | **866** | 293 | 301 | 298 | 310 | 300 |
| **Alai** | **1.068** |  | 956 | 856 | 288 | 294 | 292 | 306 | 295 |
| **Abra** | 973 | 956 |  | 857 | 287 | 292 | 289 | 303 | 294 |
| **Apal** | 866 | 856 | 857 |  | **308** | **310** | **307** | **320** | **312** |
| **Pmal** | 293 | 288 | 287 | 308 |  | 319 | 320 | 323 | 314 |
| **Paus** | 301 | 294 | 292 | 310 | 319 |  | **541** | 398 | 390 |
| **SLY** | 298 | 292 | 289 | 307 | 320 | **541** |  | 396 | 396 |
| **OY-M** | **310** | **306** | **303** | **320** | **323** | 398 | 396 |  | **448** |
| **AY-WB** | 300 | 295 | 294 | 312 | 314 | 390 | 396 | **448** |  |
| **Σ** | 1.471 | 1.379 | 1.704 | 1.441 | 497 | 839 | 1.097 | 752 | 671 |
